# Vibration-based bearing fault diagnosis in noisy conditions using matrix pencil mean frequency and multilayer perceptron neural networks

**DOI:** 10.1371/journal.pone.0345870

**Published:** 2026-03-30

**Authors:** Abderrzak Laib, Fayssal Ouagueni, Abdelghafour Herizi, Salim Djeriou, Hafiz Ahmed, Zakaria Chedjara, Saida Dahmane

**Affiliations:** 1 Department of Electrical Engineering, Faculty of Technology, LGE Research Laboratory, University of M’Sila, M'Sila, Algeria; 2 Autonex Systems Limited, London, United Kingdom; 3 School of Electrical and Electronic Engineering, The University of Sheffield, Sheffield, United Kingdom; 4 Faculty of Applied Sciences, Ibn Khaldoun University of Tiaret Algeria, Tiaret, Algeria; University of Baghdad, IRAQ

## Abstract

Bearings are critical elements in rotating machinery, where failures often accelerated by noise interference can cause severe economic and safety consequences. Reliable fault detection in noisy environments remains a major challenge. This paper proposes a novel approach combining advanced signal processing with machine learning to enhance diagnostic robustness. The matrix pencil (MP) method is applied to vibratory signals to extract matrix pencil mean frequency (MPMF) features, offering a noise-resilient spectral representation that highlights fault signatures. To improve generalization, additive white Gaussian noise (AWGN) is introduced both into the extracted features and directly into the vibratory signals, generating diverse datasets with varying signal-to-noise ratios (SNRs). This dual augmentation strategy effectively simulates real-world conditions and strengthens model resilience. A multilayer perceptron (MLP) classifier trained on the enriched feature set achieves outstanding performance, as validated on the University of Ottawa dataset (UORED-VAFCLS). The results demonstrate that the proposed method significantly enhances fault detection accuracy under noisy conditions, offering a promising solution for real-time, reliable condition monitoring in industrial applications.

## Introduction

Industrial processes continue to progress and modernize, but they always remain linked to rotating machines which play an indispensable role in production [1]. Unfortunately, these machines are impacted by several undesirable environmental factors including: high temperature, insufficient lubrication, corrosion and abrasion [2]. These factors can badly influence the working condition of the entire machine or any of its components, making the possibility of damage stronger and the disruption of productive operations is inevitable [3].

As a critical component in rotating machinery, bearings directly influence mechanical performance and account for approximately 40–50% of machine faults [4], leading to significant financial losses if not properly monitored. To prevent unexpected failures and production shutdowns, effective condition monitoring and early fault diagnosis are essential within a predictive maintenance framework that optimizes time and material resources [5,6]. Direct diagnosis is difficult due to harsh operating conditions; therefore, multiple physical quantities are monitored using sensors [7], including acoustic signals [8], thermal signals [9], lubrication parameters [10], and vibration signals [11]. Among these, vibration analysis is a primary tool for bearing fault detection in modern maintenance strategies [12]. Diagnosis is generally based on interpreting vibration signals generated during operation; however, time-domain data alone is insufficient to fully characterize bearing conditions [13,14]. Consequently, advanced signal processing approaches are employed, including time-domain, frequency-domain, and time–frequency domain analyses [13,15,16].

Temporal techniques for machinery monitoring use statistical indicators (e.g., kurtosis, crest factor), which are simple and low-cost but cannot pinpoint fault origins or enable precise diagnosis [17]. Frequency-domain analysis commonly employs FFT (fast Fourier transform); however, operational and load variations can produce non-stationary signals, limiting its effectiveness [18]. Time–frequency methods, such as empirical mode decomposition (EMD) and wavelet transform (WT), address FFT limitations. EMD adaptively decomposes signals and reduces noise without prior information but is sensitive to noise and suffers from mode mixing [19]. WT decomposes signals into sub-signals for extracting information from both low- and high-frequency bands [20,21], though its resolution limits detection to transient signals. The matrix pencil (MP) method is another effective signal-processing tool, showing strong performance with fewer limitations than many traditional techniques [22].

In practice, applying signal-processing methods for fault detection requires careful analysis and expert interpretation, including feature selection, threshold setting, and continuous monitoring, which introduces human factors that reduce reliability [23]. To address this, artificial intelligence (AI) techniques are increasingly used to automate detection and minimize human involvement. Recent studies illustrate this trend: [24] compared Random Forest, ANN, and autoencoder classifiers, with the autoencoder achieving 91% accuracy; [25] processed vibration signals in noisy environments using a CNN-based model, converting signals into images and denoising with wave atomic basis functions; [26] combined discrete wavelet transform (DWT) and ANN for motor current analysis, achieving up to 100% accuracy. These works demonstrate AI’s potential, but relying solely on raw signals is impractical in large-scale applications due to limited fault detail. Hence, effective dimensionality reduction is crucial to maintain diagnostic fidelity while reducing computational complexity. The limitations of the existing proposed approach for bearing fault diagnosis in induction motors include:

- **Environmental Sensitivity**: Rotating machines are susceptible to harsh conditions like high temperatures, poor lubrication, corrosion, and abrasion, which complicate fault detection.
- **Challenges in Direct Diagnosis**: Direct fault diagnosis is technically difficult due to the complex and varying operating conditions of bearings.
- **Limitations of Temporal Analysis:** Time-domain techniques, while simple and cost-effective, cannot precisely identify fault origins or provide detailed diagnostics.
- **Drawbacks of Frequency Domain Techniques:** FFT-based frequency analysis struggles with non-stationary signals due to operational variations, limiting its effectiveness.
- **Constraints of Time-Frequency Methods**:
- **EMD**: Though adaptive and capable of noise reduction, it is sensitive to noise and suffers from mode mixing.
- **Wavelet Transform**: Has resolution issues, making it less effective in detecting transients.
- **Manual Interpretation Dependency**: Traditional methods often require expert interpretation to analyze results, set thresholds, and perform ongoing monitoring, introducing human error and reducing system reliability.
- **Need for Dimensionality Reduction**: In AI-based methods, using raw data directly is inefficient and lacks detailed fault representation, highlighting the need for effective feature extraction and dimensionality reduction without losing critical information.
- **Lack of Real-Time Implementation**: While the approaches proposed show promise in laboratory settings or controlled environments, real-time fault detection and diagnosis in live industrial systems can be challenging due to time constraints and the need for immediate actions to prevent machine failure.

Addressing the limitations of bearing fault detection requires continuous innovation in signal processing and machine learning to achieve robust, real-time, and noise-resistant diagnostics. Traditional methods often fail under noisy conditions, emphasizing the need for adaptive, automated solutions. A recent study [27] introduced the matrix pencil mean frequency (MPMF) technique, extracting distinctive frequency features from vibration signals and achieving 100% classification accuracy with an MLP neural network on the cleaned CWRU dataset [28]. Building on this, the present work integrates the matrix pencil (MP) method with an MLP classifier for reliable fault diagnosis in noisy environments. The MP method decomposes vibration signals into frequency vectors, from which MPMF features provide a compact, noise-resilient spectral representation. To enhance robustness, additive white Gaussian noise (AWGN) is added to signals and features, creating datasets with varying SNRs that simulate realistic industrial conditions. Experiments on the University of Ottawa bearing vibration dataset [29] show consistently high classification accuracy, confirming MPMF’s discriminative power and system robustness, offering an effective solution for intelligent condition monitoring in rotating machinery. The main advantages of the proposed method are:

- **Robust Feature Extraction**: The introduction of MPMF features enhances the ability to extract meaningful fault signatures from noisy vibratory data. By averaging across signal segments, this method suppresses noise while preserving essential diagnostic information.
- **Enhanced Fault Classification Under Noise**: The MLP classifier leverages the noise-robust MPMF features, significantly improving classification accuracy. Unlike conventional approaches that degrade in performance under noise, this method ensures reliable fault detection despite signal contamination.
- **Adaptive Noise Filtering**: The MP method inherently differentiates between structural vibratory components and random noise, improving the signal-to-noise ratio. This capability enhances fault identification, particularly in environments with high background disturbances.
- **Dual Noise Augmentation Strategy:** By introducing AWGN at both the signal and feature levels, the method simulates real-world industrial noise scenarios, improving model generalization and robustness.
- **Reliable Performance in Industrial Conditions**: Designed for real-world applications, the proposed approach has been experimentally validated under noisy industrial conditions. It maintains high accuracy in fault diagnosis even when vibratory signals are affected by mechanical interference and environmental noise.
- **Detection of Developing Faults in Noisy Conditions**: The method ensures early-stage fault detection by extracting stable vibratory features that remain robust under varying noise levels. This is crucial for predictive maintenance, reducing unexpected failures and minimizing downtime in industrial machinery.
- **Consistent Monitoring in Harsh Environments**: By integrating advanced signal processing and machine learning techniques, the system ensures reliable condition monitoring, even in challenging industrial settings where noise can obscure conventional diagnostic indicators.
- **High Classification Accuracy:** The MLP classifier trained on MPMF features achieves outstanding diagnostic performance, demonstrating superior fault detection capability even at low SNRs.

Recent studies, such as the dynamic wavelet-enhanced bidirectional adaptive graph convolutional network (DyWave-BiAGCN) [30] and the dual-channel adaptive graph convolutional network (DCAGGCN) [31], have demonstrated the remarkable potential of graph neural network (GNN)-based architectures in vibration-based bearing fault diagnosis and remaining useful life (RUL) prediction. These models exploit graph structures and dynamic wavelet decomposition to capture spatiotemporal dependencies among multi-scale vibration features. While such methods achieve excellent performance in modeling complex degradation dynamics, they typically require large-scale datasets and extensive parameter tuning to maintain stability and generalization. Moreover, their deep architectures often make them sensitive to noise interference and challenging to interpret physically. In contrast, the proposed MPMF based method offers a physically interpretable, noise-resilient, and computationally efficient alternative. By directly applying the MP method to vibration signals, the MPMF feature extraction process provides a compact spectral representation that captures the dominant resonance frequencies associated with bearing faults while inherently suppressing stochastic noise components. This enables reliable fault detection even under low SNR conditions, where deep learning-based models often exhibit degradation due to their data-driven nature. Additionally, the proposed approach integrates AWGN both at the signal and feature levels, effectively simulating real-world operating variability and improving model robustness. Unlike complex GNN frameworks, the MPMF-based pipeline requires minimal computational resources and can be deployed efficiently for real-time monitoring in industrial systems. Therefore, while DyWave-BiAGCN and related GNN models excel in extracting high-level nonlinear relationships within clean and structured datasets, the proposed MPMF-based framework provides a more robust and interpretable solution for fault detection in noisy environments, aligning closely with the practical needs of predictive maintenance applications.

### Matrix pencil method overview for vibration signal analysis

#### Principles of the matrix pencil method.

The matrix pencil method (MPM) is a powerful parametric signal processing technique widely used for extracting modal parameters and spectral features from time-domain signals [32,33], especially in noisy environments. In the context of vibration signal analysis, MPM offers superior resolution and noise robustness compared to traditional Fourier-based methods. Its advantage lies in its ability to accurately estimate frequencies and damping ratios even under low Signal-to-Noise Ratio (SNR) conditions. This method has received a big attention for applications that has large frequency variations, such as shipboard power system [34]. The MP is defined as [35]:


$$\text{P}(\lambda )=\left[{\text{Y}}_{\text{2}}\right]-\lambda \left[{\text{Y}}_{\text{1}}\right],$$ (1)

where λ represents a scalar parameter and


$$\begin{array}{c} \left[{\text{Y}}_{\text{1}}\right]=[\begin{array}{ccc} \begin{array}{cc} {\text{y}}_{\text{0}} & {\text{y}}_{\text{1}}\\ {\text{y}}_{\text{1}} & {\text{y}}_{\text{2}} \end{array} & \cdots & \begin{array}{c} {\text{y}}_{\text{L}-\text{1}}\\ {\text{y}}_{\text{L}} \end{array}\\ \begin{array}{cc} \vdots & \vdots \end{array} & \ddots & \vdots \\ \begin{array}{cc} {\text{y}}_{\text{N}-\text{L}-\text{1}} & {\text{y}}_{\text{N}-\text{L}} \end{array} & \cdots & {\text{y}}_{\text{N}-\text{2}} \end{array}{]}_{(\text{N}-\text{L})\times \text{L}}\;\;\;\;\;\;\;\;\;\;\;\;\;\;\;\;\;\;\;, \end{array}$$ (2)

$$\begin{array}{c} \left[{\text{Y}}_{\text{2}}\right]=[\begin{array}{ccc} \begin{array}{cc} {\text{y}}_{\text{1}} & {\text{y}}_{\text{2}}\\ {\text{y}}_{\text{2}} & {\text{y}}_{\text{3}} \end{array} & \cdots & \begin{array}{c} {\text{y}}_{\text{L}}\\ {\text{y}}_{\text{L}+\text{1}} \end{array}\\ \begin{array}{cc} \vdots & \vdots \end{array} & \ddots & \vdots \\ \begin{array}{cc} {\text{y}}_{\text{N}-\text{L}} & {\text{y}}_{\text{N}-\text{L}+\text{1}} \end{array} & \cdots & {\text{y}}_{\text{N}-\text{1}} \end{array}{]}_{(\text{N}-\text{L})\times \text{L}}\;\;\;\;\;\;\;\;\;\;\;\;\;\;\;\;\;\;. \end{array}$$ (3)

The parameter L, known as the pencil parameter, is typically chosen between N/3 and N/2, where N is the number of exponentials modeled. The matrix pencil method represents the time-domain signal as a sum of complex exponential components. Its parametric estimation approach relies on modeling the signal as a series of damped exponentials, characterized by the model order, residual pairs, and associated poles. In the case, the modeled signal in discrete-time is given by:


$$\text{y}\left(\text{k}{\text{T}}_{\text{s}}\right)=\text{x}\left(\text{k}{\text{T}}_{\text{s}}\right)=\sum_{\text{i}=\text{1}}^{\text{i}=\text{M}}{\text{R}}_{\text{i}}\overset{\text{k}}{\underset{\text{i}}{\text{z}}}+\text{n}\left(\text{k}{\text{T}}_{\text{s}}\right),\;\text{0}\leq \text{K}\leq \text{N}-\text{1}$$ (4)

where k is the sampling instance, $T_{s}$ is the sampling time, M is the number of complex exponential signal components with i denoting the individual component, N is the total number of samples, measurement noise is denoted by n, and the complex exponentials are denoted by $R_{i}$ and $z_{i}$, respectively. These exponentials are given by [33]:


$${\text{z}}_{\text{i}}={\text{e}}^{\left({\text{s}}_{\text{i }}{\text{T}}_{\text{s}}\right)}={\text{e}}^{\left((\alpha_{\text{i }}+{\text{jw}}_{\text{i }}){\text{T}}_{\text{s}}\right)},\;{\text{R}}_{\text{i}}=\;{\text{A}}_{\text{i}}{\text{e}}^{{\text{j}\theta }_{\text{i}}},\;(\text{i}=\text{1},\text{2},\text{3}\ldots \ldots .\text{M})$$ (5)

For the *i*-th modal component, w_i_,α_i_,A_i_ , θ_i_  are the angular frequency, damping factor, amplitude and initial phase of respectively and $s_{i}$ is given by ${\text{s}}_{\text{i }}=\alpha_{\text{i }}+{\text{jw}}_{\text{i }}$. The residuals ${\text{R}}_{\text{i}}$ and the complex exponentials ${\text{z}}_{\text{i}}$ are unknowns and must be determined by the N known values of the measured signal y. These parameters can be calculated from the following equations [33]:


$$\left\{ \begin{array}{c} @l{\text{w}}_{\text{i }}=\text{Im}\left(\text{ln}\frac{{\text{z}}_{\text{i}}}{{\text{T}}_{\text{s}}}\right).\\ \alpha_{\text{i }}=\text{Re}\left(\text{ln}\frac{{\text{z}}_{\text{i}}}{{\text{T}}_{\text{s}}}\right).\\ {\text{A}}_{\text{i}}=\left|{\text{R}}_{\text{i}}\right|.\\ \theta_{\text{i}}=\text{arctan}\left[\frac{\text{im}\left({\text{R}}_{\text{i}}\right)}{\text{Re}\left({\text{R}}_{\text{i}}\right)}\right]. \end{array}\right.\;$$ (6)

The MPM and singular spectrum analysis (SSA) [36–39] are both prominent techniques in signal processing and time series analysis, yet they diverge notably in methodology, assumptions, and use cases. MPM is mainly employed for estimating the parameters such as frequencies and damping coefficients of signals composed of exponential components, especially in noisy environments. It is commonly applied in domains like system identification, modal analysis, and signal decomposition. This method conceptualizes a signal as a combination of damped or undamped exponentials, from which a Hankel or Toeplitz matrix is built. By conducting an eigenvalue decomposition of a matrix pencil, MPM extracts the key parameters of the signal. Being a parametric technique, MPM relies on a predefined model structure and typically suits applications where signals naturally exhibit exponential behavior, such as in radar, sonar, and vibration analysis. Conversely, SSA is a non-parametric framework aimed at decomposing time series data to reveal trends, periodic components, and noise. Unlike MPM, SSA does not require an assumed model form. Instead, it transforms the time series into a higher-dimensional trajectory matrix and applies singular value decomposition. The resulting components can then be interpreted individually, offering insight into different aspects of the original series, such as long-term trends or cyclical patterns. SSA finds broad application in disciplines like climatology, financial forecasting, and bioinformatics, often used for tasks like denoising, trend extraction, and feature isolation. In essence, MPM is model-based and best suited for analyzing signals with a known exponential structure, whereas SSA offers a more flexible, model-free approach that excels in exploratory analysis and feature separation. The selection of either method hinges on the data characteristics and the specific analytical objectives at hand.

### Proposed approach for vibratory signal analysis using the matrix pencil method

#### Step 1: Signal segmentation for focused analysis.

**Fig 1:**
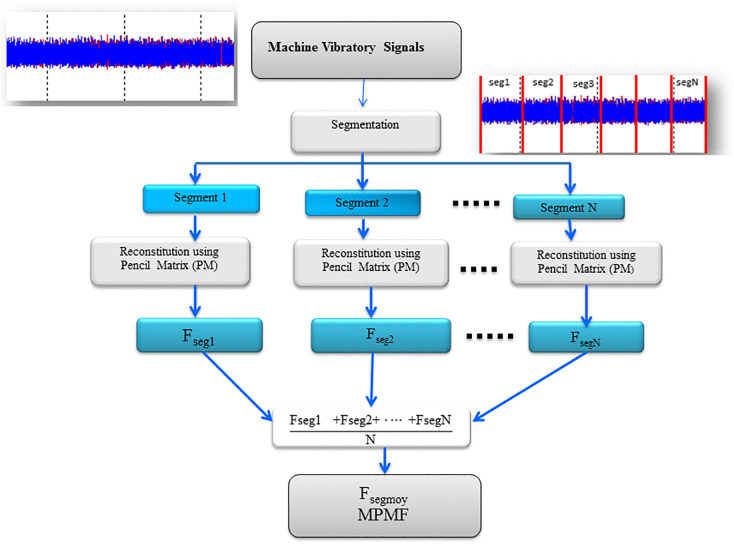
Principle of matrix pencil mean frequency extraction.

In machinery signal analysis, particularly for vibratory data obtained from accelerometers, precise segmentation is a critical preprocessing step. The vibratory signal is divided into equal-length segments (see Fig 1), ensuring a structured partitioning that enables focused analysis on discrete time intervals. This segmentation isolates distinct operational phases, facilitating the detection of anomalies or patterns indicative of wear, malfunction, or other mechanical issues. By systematically organizing the data into manageable segments, this step enhances analytical clarity, allowing for a more detailed and accurate assessment of machinery health.

#### Step 2: Matrix pencil method for signal reconstruction.

Following segmentation, the MPM is applied to extract vital information from each signal segment. Unlike traditional frequency-domain techniques such as the FFT, which may struggle with transient and non-stationary behaviors, MPM constructs structured matrices directly from time-domain data. This allows for the preservation of critical signal characteristics essential for assessing machinery health. Each segmented vibratory signal undergoes MPM processing, yielding detailed frequency vectors that encapsulate both steady-state and transient dynamics.

#### Step 3: Eigenvalue decomposition for frequency analysis.

Once the matrices are constructed, eigenvalue decomposition is performed to extract intrinsic modal frequencies and vibrational modes. This process refines the spectral analysis by identifying dominant frequencies that characterize the vibratory signature of the machinery. The resulting eigenvalue spectrum highlights peaks that correspond to essential frequency components, enabling precise identification of mechanical faults and operational irregularities. This step ensures a high-resolution frequency analysis, making it particularly useful for detecting signal components even in noisy environments.

#### Step 4: Matrix pencil mean frequency computation for comprehensive representation.

At this stage, the extracted frequency vectors from individual segments are aggregated to form a unified representation of the signal. This is achieved through an averaging process that synthesizes a MPMF vibratory vector. The MPMF vector encapsulates the collective frequency characteristics of the vibratory signal, balancing the influence of each segment and enhancing overall accuracy and resolution. This final step provides a holistic view of the signal’s dynamic behavior, significantly strengthening machinery condition monitoring, fault detection, and predictive maintenance strategies.

A bearing, an essential precision mechanical component, is designed to transform sliding friction between the shaft and bearing surface into rolling friction, thereby reducing frictional losses. It consists of four primary elements: the inner ring, the outer ring, rolling elements, and the cage, each fulfilling a unique function. The inner ring allows for alignment and rotation along the axis, while the outer ring provides support and aligns with the bearing seat. The cage maintains uniform distribution of the rolling elements between the inner and outer rings and also serves as a lubrication component [40]. Operational conditions can affect specific parts or the entire bearing, with common faults typically occurring in the inner race, cage, rolling element, and outer race. Theoretical fault frequencies for these components have been calculated and are as follows [41]:

Characteristic Frequency of Inner Ring Defects: This is the distinct vibration frequency resulting from faults in the inner ring, also known as the “bearing pass frequency of the inner race.” The formula for determining this frequency is provided below.:


$$f_{ir}=\frac{n_{b}}{\text{2}}f_{r}\left(\text{1}+\frac{d}{D}\text{cos}\alpha \right).$$ (7)

Characteristic Frequency of Cage Defects: The cage, located between the inner and outer rings, can also experience defects. These defects are characterized by a specific vibration frequency, calculated as:


$$f_{c}=\frac{\text{1}}{\text{2}}f_{r}\left(\text{1}\pm \frac{d}{D}\text{cos}\alpha \right).$$ (8)

Characteristic Frequency of Rolling Element Defects: Defects in the rolling elements occur when they come into contact with both the inner and outer rings. The resulting vibration frequency at the contact point is given by:


$$f_{re}=\frac{D}{d}f_{r}\left(\text{1}-{\left(\frac{d}{D}\text{cos}\alpha \right)}^{\text{2}}\right).$$ (9)

Frequency of Outer Ring Defects: Faults in the outer ring are identified by the vibration frequency generated as a rolling element passes through the stationary ring. This frequency, known as the “bearing pass frequency of outer race,” and is given by:


$$f_{or}=\frac{n_{b}}{\text{2}}f_{r}\left(\text{1}-\frac{d}{D}\text{cos}\alpha \right).$$ (10)

In the above formulas, ${\text{n}}_{\text{b}}$ represents the number of rolling elements (balls); d denotes the diameter of the rolling element; D signifies the bearing pitch diameter; fir, fc, fre, for denote the specific frequencies for the inner race, bearing cage, rolling element (ball), and outer race faults, respectively; and fr is the rotational frequency, which is calculated as:


$$f_{r}=\frac{n}{\text{60}}.$$ (11)

where n represents the rotation speed of the motor.

## Experimental results and discussion

### Experimental setup

The data used in this study were collected from the UORED-VAFCLS test rig [29]. While Table 1 details the motor and bearing setup, Table 2 outlines the sensor setup and data collection, and Table 3 provides information on the Experimental Setup and Data Acquisition process.

**Table 1:** Motor and bearing setup.

Component	Description
Motor	Single-phase motor mounted on a sturdy plate with anti-vibration mounts.
Shaft Adapter	Elevates the motor shaft to accommodate SKF E22206 spherical roller bearing
Bearing	SKF E22206 spherical roller bearing placed on the motor shaft to withstand the load from the beam.
Load Regulation	Load exerted by the cantilever beam is regulated via a lead screw.
Motor Speed	Constant speed of 1,750 RPM.
Internal Bearings	Two NSK 6203ZZ steel ball bearings support the motor shaft.
Drive-End Bearing Model Change	Initially used 6203ZZ bearings for 5 tests, followed by FAFNIR 203KD bearings for 15 tests.
Bearing Seal Removal	Drive-end bearing seals were removed, and bearings were degreased to accelerate deterioration.
Bearing Replacement	Bearings were replaced after each test.

**Table 2:** Sensor setup and data collection.

Component	Description
Accelerometer	PCB, model 623C01, mounted directly on the drive-end bearing via a motor casing modification.
Microphone	PCB, model 130F20, positioned 2 cm from the drive-end bearing with independent support.
Load Cell	OMEGA, model LCM302, placed between the cantilever arm and SKF E22206 bearing to transfer load.
Hall Effect Sensor	OMEGA, model OMDC-MPU-A, mounted 2 mm from a two-toothed gear on the drive shaft adapter.
Thermocouples	OMEGA, model KTSS-HH, measure both room temperature and the bearing's outer race temperature.

**Table 3:** Experimental setup and data acquisition.

Component	Description
Fault Induction	Setup allows fault induction in the drive-end bearing with minimal signal-to-noise ratio.
Accelerometer Evaluation	Mounted externally on the motor casing and internally on the bearing; internal mounting reduces noise and avoids electromagnetic interference.
Data Acquisition System	National Instruments USB-6212 connects sensors to the computer.
Signal Conditioner	PCB Piezotronics 482C used to connect accelerometer and microphone sensors.
Accelerometer Function	Detects vibration signals from the motor.
Microphone Function	Collects acoustic data.
Load Cell Function	Measures the load applied to the motor shaft.
Hall Effect Sensor Function	Tracks the rotational speed of the motor shaft.
Thermocouples Function	Monitors both room and bearing temperatures.

### Reconstructing vibratory machinery signals using pencil matrix method

**Fig 2:**
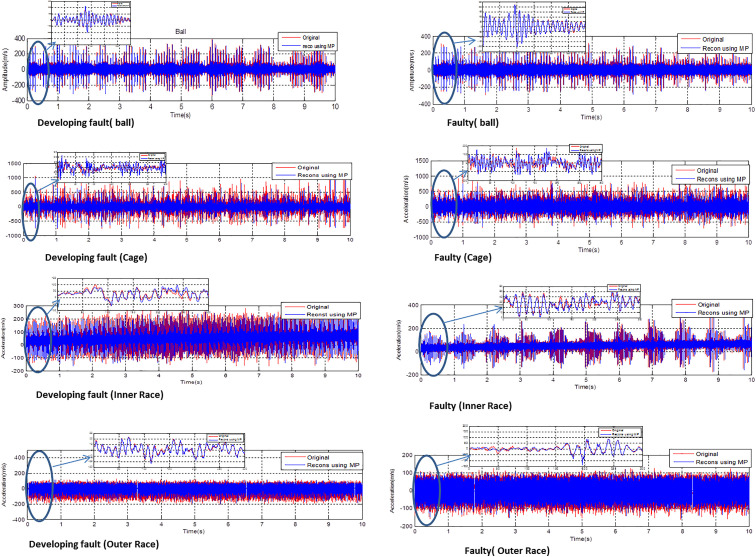
Reconstructed vibratory signal using the matrix pencil method (M = 50 poles, SL = 300).

**Fig 3:**
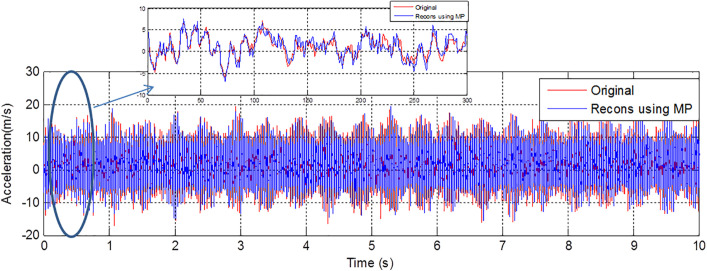
Reconstructed vibratory signal using the matrix pencil method (M = 50 poles, SL = 300) for the healthy bearing.

Figs 2 and 3 provide a comprehensive analysis of vibratory signatures and reconstituted signals using the MP across various scenarios, including healthy bearings and different fault conditions: developing and fully developed faults in the ball, cage, inner race, and outer race. The study focuses on a no-load motor example. The MPM is particularly effective for signal decomposition and reconstruction, offering a powerful tool for analyzing machinery vibrations. In this study, the MP method is applied with 50 poles and a segment length (SL) of 300 to accurately reproduce the vibratory signatures of a healthy bearing. The parameters M = 50 and SL = 300 were not selected empirically in this work. In our previous study [27], a detailed sensitivity analysis was conducted by evaluating multiple combinations of pole numbers and segment lengths. The comparative results, summarized in Table 2 of [27], showed that this configuration provided the highest classification accuracy. Therefore, these optimal values are reused here to maintain methodological continuity while focusing on the robustness analysis under noisy conditions. The method's ability to decompose complex signals into a sum of damped sinusoids allows for precise identification of various fault conditions, including those in the ball, cage, inner race, and outer race. Specifically, the MP method demonstrated its effectiveness in identifying both developing and fully developed faults by reconstituting the original vibratory signals with high fidelity. The consistent results, derived using a minimal number of poles (M = 50) and a segment length (SL = 300), underscore the method's reliability in capturing the true vibratory characteristics of machinery components under diverse conditions. These findings highlight the MPM’s robustness in faithfully reproducing original vibratory signatures, whether the bearings are healthy or exhibit various fault types. This capability is crucial for predictive maintenance and fault diagnosis in machinery.

### Enhanced analysis of vibration signals using the matrix pencil mean frequency spectrum

**Fig 4:**
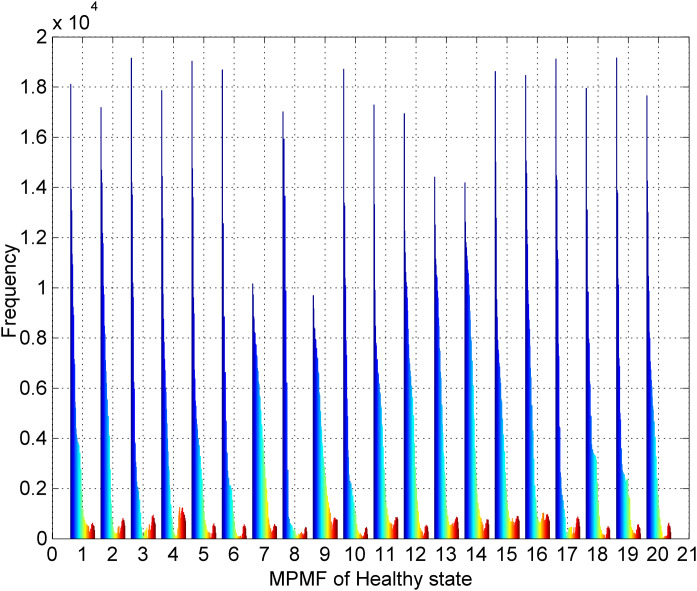
Results of the MPMF vibratory analysis for a healthy bearing state.

The use of the MPM identifies dynamic spectral components in machinery vibration signals, revealing detailed frequency variations within individual segments. By systematically combining these components, MPMF vibratory vector is created, which offers a comprehensive perspective on the signal's intrinsic characteristics. Fig 4 displays the results of the MPMF analysis for a healthy bearing state. The dataset includes twenty distinct vibration signals from healthy bearings, each reconstructed using our proposed MPM with a minimal number of poles (M = 50) and a segment length (SL = 300). Each MPMF captures the intrinsic characteristics of the healthy bearing vibration signals. The color spectrum, shifting from blue to red, represents the frequencies of the 50 poles, ranging from 1 to 50 poles. Notably, the MPMF shapes show clear distinctions, underscoring the consistency across these signals.

**Fig 5:**
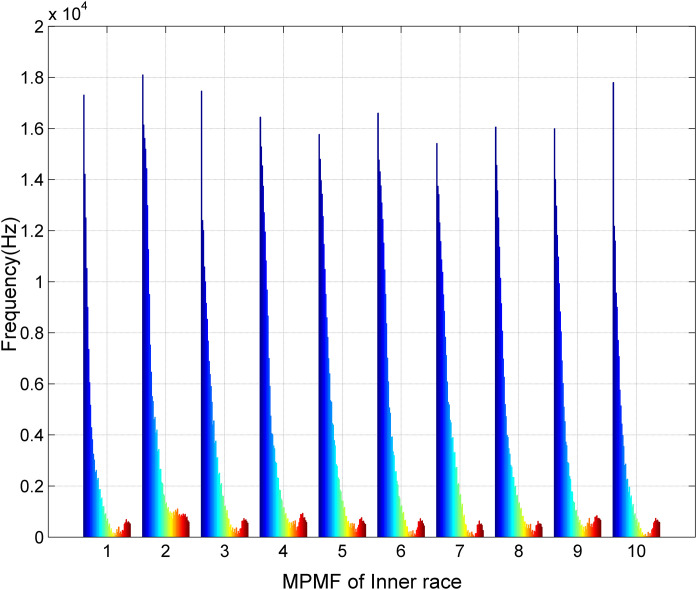
Results of the MPMF vibratory analysis for bearings with an inner race fault.

Fig 5 illustrates the second scenario of MPMF vibratory analysis for bearings with an inner race fault. The dataset comprises ten distinct vibration signals from bearings with inner race faults: five from bearings with a developing fault and five from bearings with a fully developed fault. Each signal was reconstructed using our MP method with a minimal number of poles (M = 50) and a segment length (SL = 300). The MPMFs encapsulate the intrinsic characteristics of the faulty bearing vibration signals. The color spectrum, transitioning from blue to red, represents the frequencies of the 50 poles, ranging from 1 to 50.The first MPMF corresponds to the first bearing with a developing inner race fault, and the second to the first bearing with a fully developed inner race fault. This pattern continues with the third and fourth MPMFs representing the second bearing, the fifth and sixth representing the third bearing, the seventh and eighth representing the fourth bearing, and the ninth and tenth representing the fifth bearing. The distinct shapes of the MPMFs highlight the consistency across these signals and allow for clear differentiation between the developing and fully developed fault cases.

**Fig 6:**
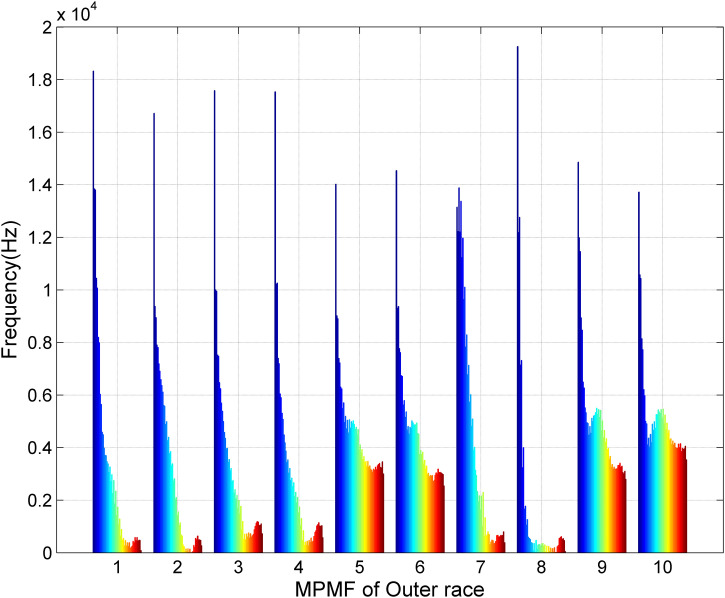
Results of the MPMF vibratory analysis for bearings with an outer race fault.

Fig 6 presents the third scenario of MPMF vibratory analysis for bearings with an outer race fault. This dataset includes ten unique vibration signals from bearings with outer race faults: five representing bearings with a developing fault and five representing bearings with a fully developed fault. Each signal was reconstructed using our MP method, utilizing 50 poles (M = 50) and a segment length of 300 (SL = 300). The MPMFs capture the essential characteristics of the faulty bearing vibration signals. The color spectrum, ranging from blue to red, indicates the frequencies of the 50 poles. The first MPMF depicts the first bearing with a developing outer race fault, while the second represents the same bearing with a fully developed fault. This sequence continues with the third and fourth MPMFs corresponding to the second bearing, the fifth and sixth to the third bearing, the seventh and eighth to the fourth bearing, and the ninth and tenth to the fifth bearing. The distinct MPMF shapes emphasize the consistency across these signals, allowing for clear differentiation between the developing and fully developed fault conditions.

**Fig 7:**
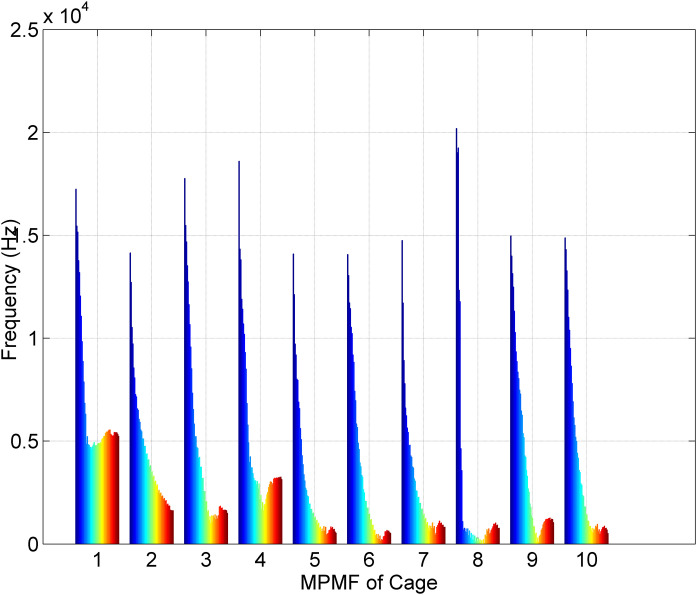
Results of the MPMF vibratory analysis for bearings with a cage fault.

Fig 7 showcases the fourth scenario of MPMF vibratory analysis for bearings with a cage fault. This dataset consists of ten unique vibration signals from bearings with cage faults: five from bearings with a developing fault and five from bearings with a fully developed fault. Each signal was reconstructed using our MP method, employing 50 poles (M = 50) and a segment length of 300 (SL = 300). The MPMFs highlight the key characteristics of the faulty bearing vibration signals. The color spectrum, transitioning from blue to red, represents the frequencies of the 50 poles. The first MPMF illustrates the first bearing with a developing cage fault, while the second shows the same bearing with a fully developed fault. This pattern continues with the third and fourth MPMFs representing the second bearing, the fifth and sixth representing the third bearing, the seventh and eighth representing the fourth bearing, and the ninth and tenth representing the fifth bearing. The distinct MPMF shapes underscore the consistency across these signals, enabling clear differentiation between the developing and fully developed fault conditions.

**Fig 8:**
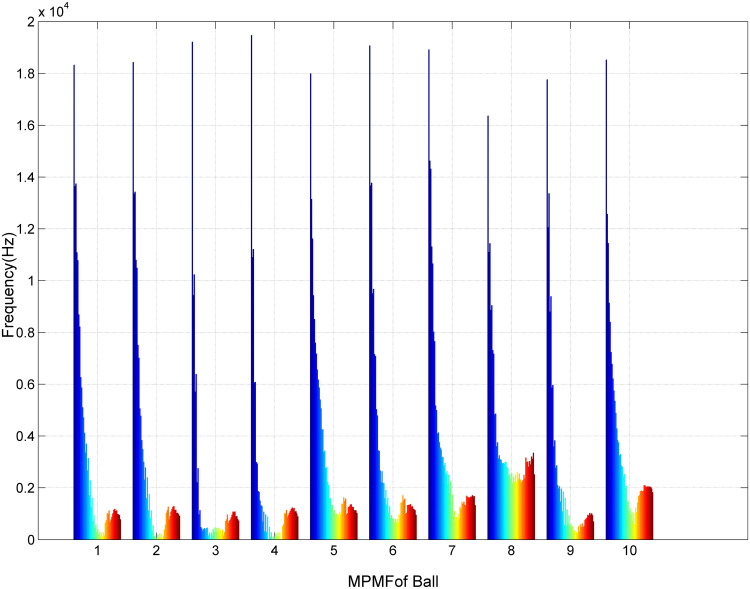
Results of the MPMF analysis for bearings with a ball fault.

Fig 8 presents the fifth scenario of MPMF vibratory analysis, focusing on bearings with ball faults. The dataset includes ten unique vibration signals from bearings: five from bearings with a developing fault and five from bearings with a fully developed fault. Each signal was reconstructed using the MP method, with 50 poles (M = 50) and a segment length of 300 (SL = 300). The MPMFs capture the key characteristics of the faulty bearing vibration signals. The color spectrum, ranging from blue to red, represents the frequencies of the 50 poles. The first MPMF depicts the first bearing with a developing ball fault, while the second represents the same bearing with a fully developed fault. This sequence continues with the third and fourth MPMFs corresponding to the second bearing, the fifth and sixth to the third bearing, the seventh and eighth to the fourth bearing, and the ninth and tenth to the fifth bearing. The distinct shapes of the MPMFs highlight the consistency across these signals, allowing for clear differentiation between the developing and fully developed fault conditions.

**Fig 9:**
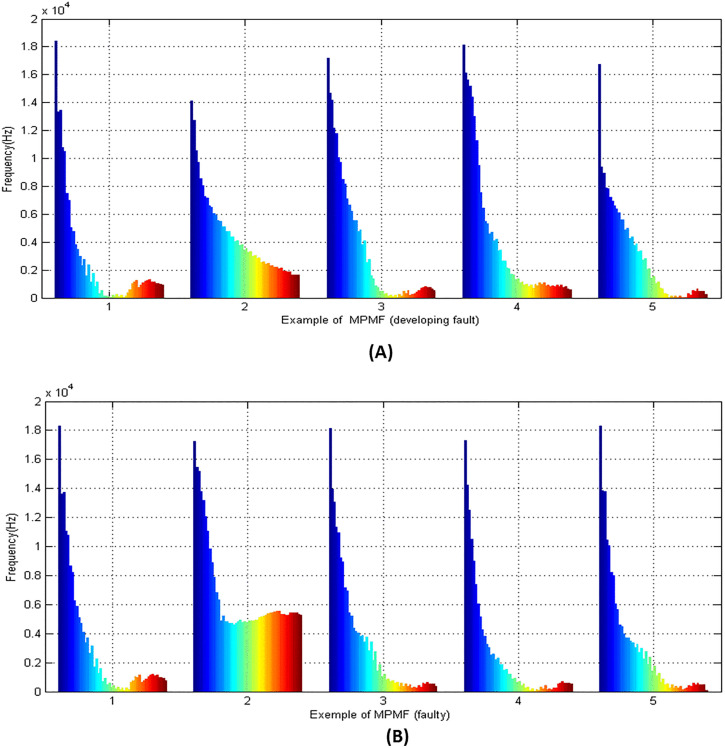
Examples of MPMF vibratory shapes corresponding tovarious bearing conditions, including developing and fully developed faults.

Fig 9 presents a series of MPMF vibratory shapes corresponding to different bearing conditions, illustrating both developing and fully developed faults. In Fig 9(A), the shapes represent bearings with developing faults, while Fig 9(B) shows the corresponding shapes for fully developed faults. Specifically, the first shape in each figure corresponds to a ball fault; developing in (A) and fully developed in (B). The second shape represents a cage fault in its developing stage in (A) and fully developed stage in (B). The third shape depicts a healthy bearing, which remains consistent across both figures and serves as a baseline reference. The fourth shape corresponds to an inner race fault developing in Fig 9(A) and fully developed in Fig 9(B). Similarly, the fifth shape illustrates an outer race fault in both its developing and fully developed states across the two figures. Each fault type exhibits a distinct MPMF spectrum shape, effectively capturing the vibrational characteristics specific to the fault type and severity. These unique spectral profiles demonstrate the capability of the MPMF technique to distinguish between various bearing conditions with clarity and precision.

### Data augmentation of vibratory signals via the matrix pencil mean frequency method

Intelligent fault diagnosis with small and imbalanced data involves developing intelligent diagnosis models using a limited number of machine fault samples to achieve accurate fault identification. Generally, intelligent diagnosis models based on deep networks rely heavily on comprehensive machine monitoring data analysis [42]. The greater the volume of training data and the more diverse the fault types included in the training set, the higher the diagnostic accuracy of these models. However, creating an ideal dataset for training intelligent diagnosis models is challenging in real-world engineering scenarios. Data-driven intelligent fault diagnosis has garnered significant attention and extensive study. Research findings have shown that these models typically exhibit high diagnostic performance [43]. However, in practical engineering scenarios, the collection of machine fault samples poses a considerable challenge, limiting the application of data-driven intelligent diagnosis models. To address this issue, data augmentation has emerged as an effective technique to enhance the generalization performance of neural networks [44]. Methods such as data generation with limited faulty data [45,46], data over-sampling [47,48], and data reweighting [49,50] have been proposed to expand the limited fault datasets, thereby enabling more effective training of intelligent diagnosis models. Consequently, these models are anticipated to maintain robust diagnostic capabilities even when fault data samples are scarce. Recently, data generation models, such as generative adversarial networks (GAN) [51] and variational auto-encoders (VAE) [52], have been extensively studied and have demonstrated promising results in various fields [53]. These generative models can also be applied to generate mechanical signals, offering a potent tool for data augmentation in small and imbalanced intelligent fault diagnosis (S&I-IFD) [54].

### Augmentation by direct noise injection into MPMF features

**Fig 10:**
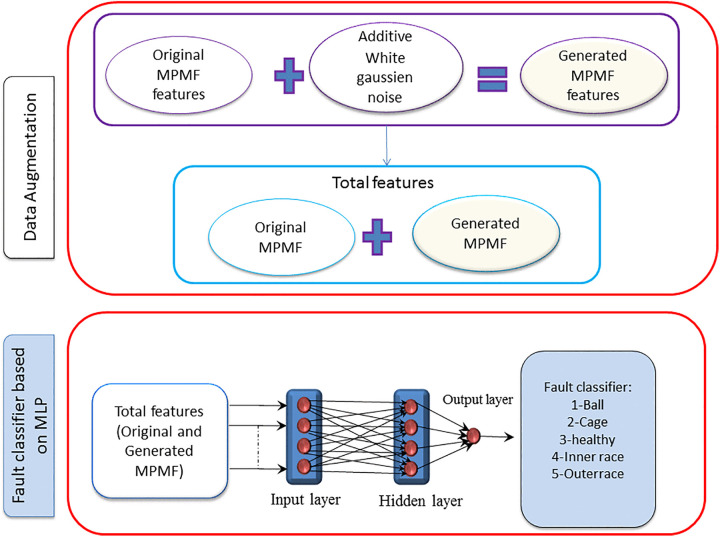
Proposed method for introducing additive white Gaussian noise to each MPMF vector to produce new MPMF vectors.


In this paper, data augmentation is implemented using the MPMF Method for vibratory signals, where AWGN is added to each of the MPMF vectors (Fig 10) to generate a new MPMF. The SNR for MPMF is set to −10 dB,-5 dB,1 dB and 10 dB. The power of the noise, denoted as $P_{n}$, is related to the power of MPMF ($P_{s}$) as given below:

$$SNR=\text{1}\text{0}\text{log}(\frac{P_{s}}{P_{n}}).$$ (12)

The original database consists of 60 MPMF vectors. After adding AWGN, the database expands to 120 examples, comprising 60 original MPMF vectors and 60 generated MPMF vectors with added AWGN. The database, therefore, contains a total of 120 examples, which are randomly divided for training and validation purposes. Approximately 80% of these examples are allocated for training, with the remaining 20% reserved for validation. Considering various SNR at levels of −15 dB, −10 dB, −5 dB, 1 dB,5dB,6dB and 10 dB, the number of databases increases to seven. Consequently, seven databases are employed for training and validating the multilayer perceptron (MLP). The evaluation of fault classification performance is quantified using metrics such as accuracy, sensitivity, specificity, precision, and G-mean.

The seven datasets used in this study consist of examples that relate MPMF features with fault locations, including healthy cases (undamaged), outer race faults, inner race faults, and rolling element faults, including cage faults. The generalization capability is assessed using the root mean square (RMS) error. These datasets have been employed for training and validating the MLP classifier [55–56].

Each dataset contains MPMF vectors generated under specific conditions, including segment length and the number of poles. In our study, the segment length is set to SL = 300, while the number of poles is M = 50. Fault classification performance is evaluated using metrics such as accuracy, sensitivity, specificity, precision, and g-mean. Accuracy, which represents the proportion of correctly classified examples, and is mathematically expressed as:


$$Acc=\frac{TP+TN}{TP+TN+FP+FN}.$$ (13)

Table 4 summarizes the performance of the MLP classifier across seven databases corresponding to different Signal-to-Noise Ratio (SNR) levels. The evaluation metrics include Accuracy, Sensitivity, Specificity, Precision, and G-mean. The original dataset consists of 60 MPMF feature vectors. After augmentation using additive white Gaussian noise (AWGN), the dataset is expanded to 120 samples (60 original and 60 noise-corrupted MPMF vectors). The augmented dataset is randomly divided into 80% for training and 20% for validation.

**Table 4:** Performance summary of MLP classifier across seven different datasets.

	Evaluation criteria				
Databases(SL,M)	Accuracy	Sensitivity	Specificity	Precision	G-mean
**Database 1** **(−15 dB)**	100	100	100	100	100
**Database 2** **(−10 dB)**	83.33	63.63	100	100	79.77
**Database 3** **(−5 dB)**	91.66	50	100	100	70.71
**Database 4** **(1dB)**	100	100	100	100	100
**Database 5** **(5dB)**	100	100	100	100	100
**Database 6** **(6dB)**	100	100	100	100	100
**Database 7** **(10dB)**	100	100	100	100	100

The results demonstrate that the proposed MPMF–MLP framework maintains strong diagnostic capability under varying noise conditions. At SNR = −15 dB, the classifier achieves 100% across all evaluation metrics. This indicates that the MPMF feature extraction effectively preserves discriminative fault information even under severe noise contamination.

**Fig 11:**
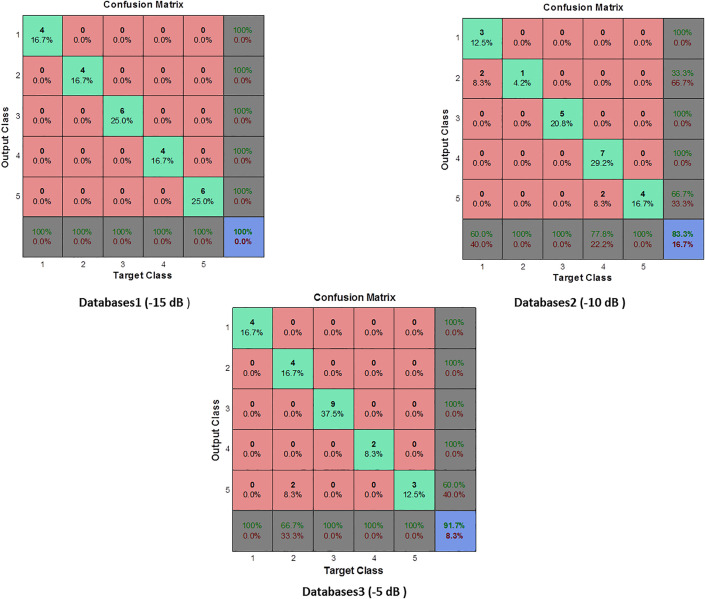
Confusion matrix for MLP classifier based on MPMF (Database cases 1, 2, 3).

At SNR = −10 dB, the overall accuracy decreases to 83.33%, primarily due to a reduction in Sensitivity (63.63%), while Specificity and Precision remain at 100%. This behavior suggests that the classifier remains highly reliable in avoiding false alarms (no false positives), but some developing fault samples are misclassified under increased noise influence. Similarly, at SNR = −5 dB, the model achieves 91.66% Accuracy with perfect Specificity and Precision, while Sensitivity decreases to 50%, leading to a G-mean of 70.71%. This indicates that although the system maintains excellent capability in correctly identifying healthy conditions and confirmed faults, the detection of developing faults becomes more challenging as noise increases. Overall, these results confirm that the proposed MPMF-based framework exhibits strong robustness to noise (Fig 11), with performance degradation occurring gradually and predictably as SNR decreases.

**Fig 12:**
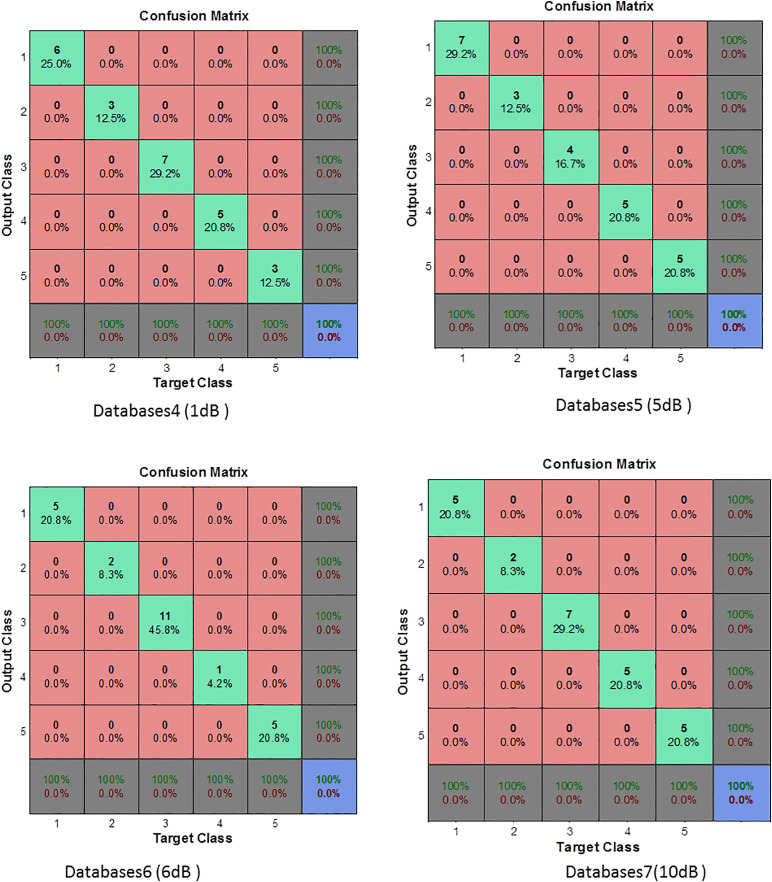
Confusion matrix for MLP classifier based on MPMF (Database cases 4, 5, 6, 7).

In contrast, when the SNR increases to 1 dB, 5 dB, 6 dB, and 10 dB (Fig 12), the MLP classifier achieves 100% across all evaluation metrics, including Accuracy, Sensitivity, Specificity, Precision, and G-mean. This perfect performance indicates that, under low-noise conditions, the extracted MPMF features provide highly separable representations of healthy and faulty classes, enabling the classifier to distinguish both true positives and true negatives without misclassification. The observed trend reveals that performance degradation is not monotonic with respect to SNR. While the classifier maintains robustness even at very low SNR (−15 dB), moderate noise levels (−10 dB and −5 dB) introduce partial ambiguity, primarily affecting Sensitivity. This behavior suggests that developing fault signatures may overlap with noise components at intermediate SNR values, leading to missed detections despite the preservation of high Specificity and Precision. As the noise level decreases further (positive SNR values), class separability improves significantly, resulting in complete recovery of diagnostic performance. The incorporation of AWGN during data augmentation plays a critical role in this behavior, as it exposes the classifier to a wide range of noise conditions during training, thereby enhancing its generalization capability. Consequently, the proposed MPMF–MLP framework demonstrates stable and reliable fault detection performance across both noisy and near-ideal operating environments.

### Enhancing robustness through noise injection in the original dataset: A case study on noisy environments

**Fig 13:**
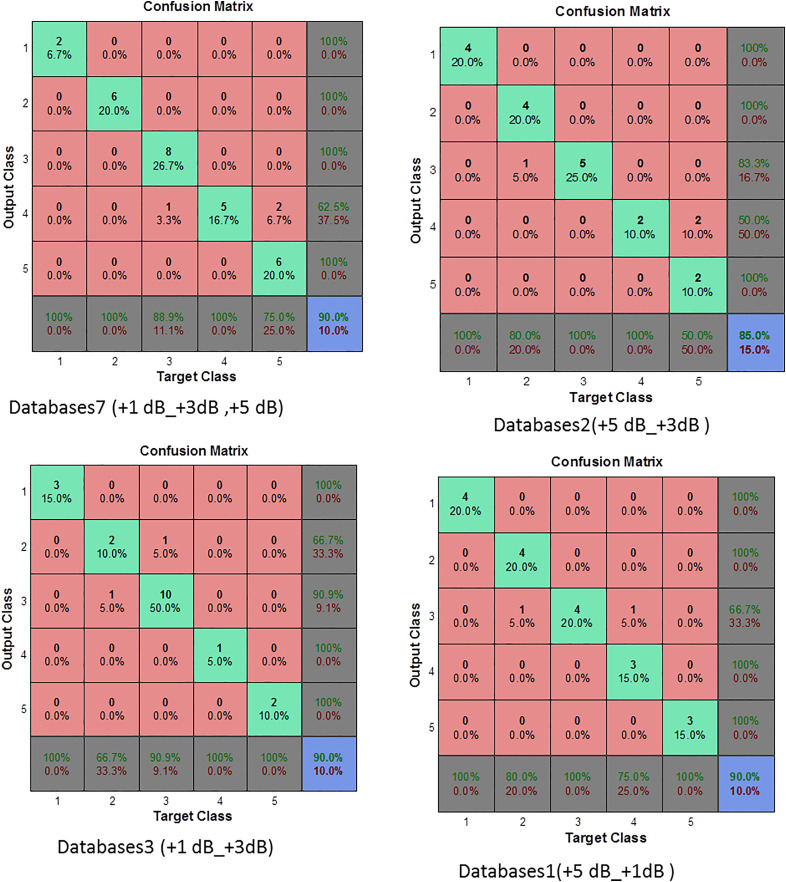
Confusion matrix for MLP classifier based on MPMF (Database cases 1, 2, 3, 7).

**Fig 14:**
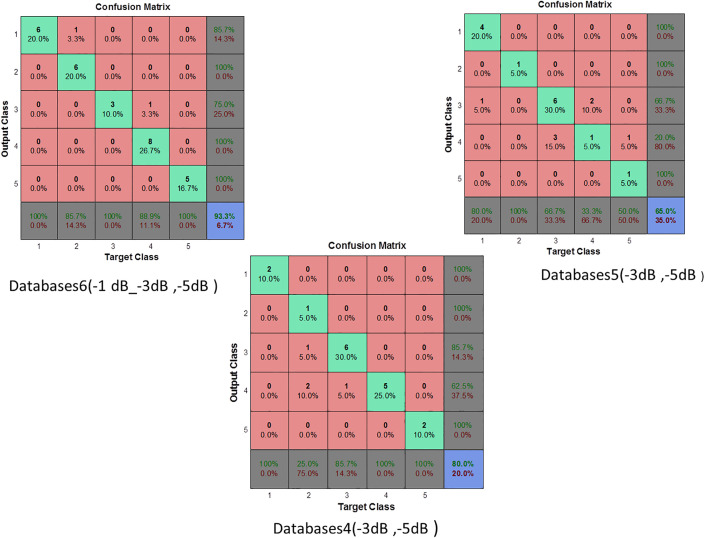
Confusion matrix for MLP classifier based on MPMF (Database cases 4, 5, 6).

In the previous section, the considered database was made with only one SNR level. Here, we made a more detailed analysis by creting databases of different noise levels at the same time. The considered databases are given in Table 5. The variations detailed in Table 5 provide a comprehensive framework for testing the performance of the proposed method under a wide range of realistic environments. Figs 13 and 14 demonstarte the performance of the proposed method evaluated across seven database configurations as detailed in Table 5.

**Table 5:** Classification results of the MLP model across seven datasets under various noisy environment conditions.

	Evaluation criteria				
Databases (SL,M)	Accuracy	Sensitivity	Specificity	Precision	G-mean
**Databases1** **(+5 dB_ + 1dB)**	90	60	100	100	77.46
**Databases2** **(+5 dB_ + 3dB)**	85	66.66	88.23	0.5	57.73
**Databases3** **(+1 dB_ + 3dB)**	90	33.33	100	100	57.73
**Databases4** **(−1 dB_-3dB)**	80	83.33	78.57	62.5	72.16
**Databases5** **(−3 dB_-5dB)**	65	25	75	20	22.36
**Databases6** **(−1 dB_-3dB,-5 dB)**	93.33	80	100	100	89.44
**Databases7** **(+1 dB_ + 3dB, + 5 dB)**	90	100	88	62.5	79.05

The evaluation was based on key performance metrics accuracy, sensitivity, specificity, precision, and G-mean to assess the model’s robustness in different noise scenarios. In Databases1 (+5 dB, + 1 dB), the MLP classifier achieved 90% accuracy, with perfect specificity (100%) and precision (100%), indicating a strong ability to identify negative cases correctly. However, sensitivity was 60%, showing that the model struggled to detect some positive cases. This may be due to the mismatch in noise levels between the training and testing sets, which affects the model’s recall performance.

In Databases2, accuracy dropped slightly to 85%, while sensitivity (66.66%) improved compared to Databases1. However, precision (50%) and G-mean (57.73%) were significantly lower, suggesting that while the model better detected positives, it did so at the expense of increased false positives possibly because the test data was noisier than the training set. Databases3 maintained high accuracy (90%), and both specificity and precision reached 100%, but sensitivity dropped to 33.33%, again indicating a bias toward the negative class. The G-mean remained at 57.73%, underlining the imbalance in class-level performance, likely because the MLP was trained on slightly cleaner data than it encountered during testing. A more diversified training set was used in Databases7, which helped the model achieve 90% accuracy and 100% sensitivity, with a G-mean of 79.05%. However, the specificity dropped to 88% and precision to 62.5%, reflecting a shift toward over-detecting positives, which is typical when the model is trained with more varied but predominantly clean signals.

Databases4 provided a useful scenario for evaluating the model under moderate noise. The MLP achieved 80% accuracy, with balanced sensitivity (83.33%) and specificity (78.57%), along with a fair G-mean of 72.16%. The precision of 62.5% confirms a reasonable trade-off between false positives and false negatives, indicating good generalization to similarly noisy environments. In more challenging conditions such as Databases5, where both training and testing data were severely noisy, the MLP’s performance degraded significantly. It reached only 65% accuracy, with 25% sensitivity, 20% precision, and the lowest G-mean (22.36%). These results highlight the MLP’s limitations when trained and tested under high-noise conditions without sufficient noise variation in training. Conversely, the best performance was observed in Databases6, where the MLP was trained with a broad range of noisy data. It achieved 93.33% accuracy, 80% sensitivity, 100% specificity and precision, and the highest G-mean (89.44%) among all configurations. This clearly demonstrates that exposure to a variety of noisy conditions during training enables the MLP to generalize more effectively to similarly complex environments. Based on the results presented in Table 4 and Figs 12 and 13, the MLP neural network showed high classification accuracy when supported by robust feature extraction through MPMF, but its performance was strongly influenced by the noise conditions in both the training and testing sets. Models trained on narrowly defined or cleaner datasets tended to struggle with sensitivity under mismatched or noisier conditions. In contrast, training on diversified noise levels, as in Databases6 and 7, allowed the MLP to achieve better generalization, especially in terms of G-mean, sensitivity, and precision, crucial factors for real-world fault diagnosis tasks.

A comparative assessment between the two noise injection strategies feature-level and signal-level augmentation was conducted to evaluate their individual effects on classification performance. When noise was injected directly into the MPMF features (Table 4), the MLP classifier consistently achieved near-perfect results, with 100% accuracy, sensitivity, specificity, precision, and G-mean across most SNR levels. Only a slight reduction was observed at –10 dB and –5 dB, where accuracy decreased to 83.33% and 91.66%, respectively. This indicates that the feature-level augmentation effectively enhances noise robustness by preserving the discriminative characteristics of the MPMF features even under severe noise interference. In contrast, when noise was applied to the vibration signals prior to feature extraction (Table 5), the classifier’s performance exhibited greater variability. While acceptable accuracy (≥ 90%) was maintained under moderate-to-high SNR conditions (e.g., + 1 dB to +5 dB), a noticeable degradation occurred in low-SNR environments, such as the –3 dB_–5 dB case, where accuracy dropped to 65% and G-mean to 22.36%. This behavior can be attributed to the distortion introduced at the signal level, which affects the precision of the extracted MPMF features. Comparatively, the results confirm that feature-level noise injection provides superior resilience against noise, whereas signal-level augmentation enhances the generalization capacity of the classifier by simulating realistic operating conditions. Therefore, the integration of both strategies within the proposed dual augmentation framework allows the MPMF–MLP model to benefit from the complementary strengths of each method, achieving robust and reliable fault detection across diverse noise scenarios. Although the present validation focuses on the University of Ottawa bearing dataset (UORED-VAFCLS), this selection ensures controlled experimental conditions with precisely defined fault categories and adjustable noise levels, which are crucial for assessing the proposed dual augmentation strategy. It is worth noting that the same MPMF-MLP framework has already been successfully validated on the Case Western Reserve University (CWRU) bearing dataset in a recent study [27]. The findings from that work confirmed the general applicability of the proposed approach to different bearing datasets. Therefore, the current study extends the previous validation by emphasizing robustness under varying SNR conditions and further demonstrating the adaptability of the proposed method to real-world industrial noise environments.

### Benchmarking against existing approaches

All results in Tables 6 and 7 were obtained using the Case Western Reserve University (CWRU) [28] bearing dataset. Our proposed MPMF-MLP approach is based on the UORED-VAFCLS framework) [29].

**Table 6:** Classification accuracies (%) on the CWRU bearing dataset [57,58].

Method	Classifier	Accuracy (%)
WPT + LDA	LDA	100
WPT + Random Forest	RF	96.22
WPT + K-Nearest Neighbors	KNN	83.79
WPT + SVM (Gaussian Kernel)	SVM	76.43
WPT + Boruta Feature Selection + KNN	KNN	97.06
EMD + LSTM	LSTM	98
**Proposed MPMF-MLP (UORED-VAFCLS)**	**MLP**	**100**

**Table 7:** Comparison of signal-processing and machine-learning techniques for fault detection.

Technique/Model	References	Signal Types Used	Performance/Results	Conditions
Convolutional neural networks (CNNs)	[59]	Stator current + vibration signals	96% detection efficiency using both signals; 50% with one signal	Rolling bearings in PMSM drives
Deep Autoencoder	[60]	Stator current + vibration signals	91% accuracy (noise-free); 88% (with noise); current-only: 99%/75%; vibration-only: 69%/66%	Classifying undamaged, outer-race faults, and 10% inter-turn short-circuit faults
Support Vector Machine with Gaussian RBF Kernel	[61]	Stator current + mechanical vibration signals	93% classification accuracy	PMSM motors under varying speed and load-torque conditions
Multilayer Perceptron (MLP)	[62]	Stator current data	100% detection efficiency under demanding load and speed conditions	Eccentricity and rolling bearing damage in PMSM
Self-Organizing Maps (SOMs) / Kohonen Neural Networks (KNNs)	[63]	Vibration signals (FFT + wavelet transforms)	Minimal topological error (±0.04) and moderate quantization error (±0.5)	Fault classification across five loading scenarios, five speeds, and eight fault types
**Proposed Matrix Pencil Mean Frequency /MLP (UORED-VAFCLS)**	Proposed	Vibrations signals	93.33% accuracy in noisy environments	Four fault types across three health states under constant load/speed

Table 6 presents a comparative evaluation of various signal processing and classification methods for bearing fault diagnosis using the CWRU dataset. Traditional techniques, such as wavelet packet transform (WPT) combined with linear discriminant analysis (LDA), achieve perfect classification accuracy (100%), while other combinations such as WPT with random forest (96.22%) and Boruta feature selection with K-Nearest Neighbors (97.06%) also demonstrate strong performance. Deep learning approaches like empirical mode decomposition (EMD) followed by an LSTM classifier further improve generalization, achieving 98% accuracy. However, performance varies notably across different classifier choices, as seen with WPT-SVM (76.43%) and WPT-KNN (83.79%), highlighting the sensitivity of these pipelines to both feature extraction and classification components. In contrast, the proposed MPMF-MLP method, developed using the UORED-VAFCLS dataset, achieves 100% classification accuracy, matching the best traditional methods and outperforming the deep learning baseline. Crucially, this result is obtained under noisy conditions, where noise is directly added to the MPMF features, unlike the other approaches, which are typically evaluated in clean environments. This clearly highlights the robustness and reliability of the proposed method, demonstrating its strong potential for real-world industrial applications where sensor noise and signal contamination are unavoidable.

In noisy environments, the proposed MPMF Vibration/MLP approach achieved an accuracy of 93.33%, outperforming the Deep Autoencoder method [60], which reported 88% accuracy under similar noise conditions. This highlights the superior robustness of the proposed MPMF method in extracting meaningful fault-related features from vibration signals even under significant noise interference. Other techniques listed in Table 7 demonstrate strong performances but under different circumstances. CNNs [59] achieved 96% detection efficiency when combining vibration and current signals, but their performance dropped considerably when using only one type of input, emphasizing their reliance on multisensory data fusion. The MLP method [62] maintained 100% detection efficiency, but its evaluation was performed under noise-free conditions, limiting its proven resilience to real-world disturbances. Similarly, the SVM with Gaussian RBF Kernel [61] showed good classification capability under varying speeds and load torques, demonstrating adaptability to dynamic operating conditions rather than noisy environments. Meanwhile, self-organizing maps (SOMs) [63] achieved low topological and quantization errors across a wide range of fault types, showcasing versatility but without direct evaluation in high-noise scenarios. Taken together, the results on the CWRU dataset demonstrate that the UORED-VAFCLS-based MPMF-MLP method consistently matches or outperforms all benchmark techniques. Notably, the model achieves 100% classification accuracy even when noise is directly injected into the MPMF features, and maintains a strong 93.33% accuracy when noise is applied to the raw vibration signals prior to feature extraction. These findings clearly highlight the robustness and effectiveness of the matrix-pencil approach under challenging and noisy conditions.

Although deep learning architectures such as CNNs and transformer-based models have shown strong performance in large-scale signal classification tasks, their application is not suitable in the present context. The available dataset, even after augmentation, contains only 120 samples, which is insufficient for reliable deep learning training without severe overfitting. Moreover, the proposed method relies on Matrix Pencil Mean Frequency (MPMF) features, which already provide a compact and highly discriminative representation of the vibration signal. In this framework, the classifier operates on informative parametric features rather than raw signals, making a lightweight MLP classifier more appropriate. Finally, the objective of this work is to develop a method compatible with real-time industrial monitoring systems with low computational requirements. Deep learning models would significantly increase complexity and computational cost without providing proportional benefits in this feature-based framework.

#### Computational requirements and real-time feasibility of the MPMF extraction.

For vibration-based condition monitoring systems intended for industrial deployment, the computational burden and real-time capability of the signal processing method are critical considerations. The proposed approach relies on the Matrix Pencil Method (MPM) to extract the Matrix Pencil Mean Frequency (MPMF) features from noisy vibration signals. Therefore, it is important to analyze the computational requirements of this stage. In the proposed implementation, the MPM algorithm operates on short vibration signal segments with a segment length SL = 300 samples. The method involves the construction of two Hankel matrices followed by a Singular Value Decomposition (SVD), which represents the most computationally demanding operation of the algorithm. However, due to the small segment length and the choice of the pencil parameter, the Hankel matrices remain of moderate size. Moreover, only M = 50 significant poles are retained for the MPMF computation, which further reduces the processing requirements. As a result, the overall computational load of the feature extraction process remains low.

To quantify the processing time, practical measurements were performed using MATLAB on a standard computer equipped with an Intel i7 processor and 16 GB RAM. The average time required to extract the MPMF feature from one vibration signal segment was found to be on the order of a few milliseconds. This processing time is significantly shorter than the signal acquisition window, meaning that feature extraction can be completed before the next segment is acquired.

These results demonstrate that the MPMF extraction process is sufficiently fast to satisfy real-time monitoring constraints. In addition, since each segment is processed independently, the algorithm is well suited for parallel implementation or deployment on embedded processors, DSPs, or FPGA-based monitoring systems.Therefore, the proposed MPMF-based diagnostic approach is not only robust to noise but also computationally efficient and fully compatible with real-time vibration-based bearing condition monitoring.

#### Validation strategy and class imbalance handling.

To ensure reliable performance estimation and avoid biased evaluation due to class imbalance and limited original samples, a stratified 5-fold cross-validation procedure was applied on two datasets:

- 60 original MPMF vectors + 60 noise-augmented vectors at **SNR = +5 dB**
- 60 original MPMF vectors + 60 noise-augmented vectors at **SNR = −5 dB**

Each dataset therefore contains **120 samples**. The results are summarized in Table 8.

**Table 8:** 5-Fold cross-validation results under different noise levels.

**Dataset**	**Accuracy**	**Sensitivity**	**Specificity**	**Precision**	**G-mean**
**Original + SNR + 5 dB**	91.67% ± 5.89%	70.00%	100%	100%	83.13%
**Original + SNR −5 dB**	**86.67% ± 7.45%**	**61.67%**	**95.78%**	**86.67%**	**70.14%**

To ensure statistical reliability of the reported results, a stratified 5-fold cross-validation procedure was employed, and all performance metrics are reported as mean ± standard deviation across the folds.The results show that the proposed MPMF-MLP framework maintains stable performance across folds and remains effective even under very severe noise conditions (−5 dB). The natural decrease in sensitivity under extreme noise confirms the realism of the evaluation and demonstrates the robustness of the proposed approach.

## Discussion and strengths of the proposed approach

The proposed method presents a robust and innovative framework for bearing fault diagnosis under noisy conditions by integrating the matrix pencil method (MPM) for feature extraction with a multilayer perceptron (MLP) classifier. The strength of this work lies in its ability to extract highly discriminative vibration features that remain stable and reliable even when the signal is heavily contaminated by noise. Unlike traditional transforms that are sensitive to noise and parameter tuning, the MPM-based mean frequency (MPMF) features effectively isolate the dominant vibratory modes, thereby preserving essential fault-related information while suppressing stochastic disturbances. A key innovation of this study is the dual noise augmentation strategy, where additive white Gaussian noise (AWGN) is introduced at both the signal and feature levels. This approach not only enhances data diversity but also significantly improves model robustness and generalization in realistic industrial environments. The comparative analysis confirmed that both augmentation mechanisms contribute positively to classifier performance, validating the necessity of their combined use. Furthermore, the study highlights the adaptive noise filtering property of the MPMF, which inherently distinguishes between structural vibration components and random noise. This capability provides a substantial improvement in the signal-to-noise ratio and leads to more accurate fault detection, even at low SNR conditions. The MLP classifier built upon these noise-resilient features achieved high classification accuracy and stable diagnostic performance, demonstrating the effectiveness of the proposed approach in realistic and challenging operating environments.

Although the experimental validation focused on the University of Ottawa bearing dataset, this choice ensures rigorous evaluation under controlled conditions with well-characterized faults. In addition, the proposed method has also been validated with the CWRU dataset in previous work [27], confirming its consistency and reliability across different databases. The results collectively indicate that the proposed MPM–MLP framework not only enhances diagnostic precision but also provides a generalizable and computationally efficient solution for industrial fault detection using vibration signals.

## Conclusion

This study proposed a novel and effective methodology for machinery fault detection and condition monitoring by combining the matrix pencil method (MPM) for feature extraction with a multilayer perceptron (MLP) neural network for classification. Unlike traditional approaches such as wavelet packet transform (WPT) and empirical mode decomposition (EMD), the MPM directly extracts frequency components from vibratory signals without relying on predefined basis functions or suffering from mode mixing issues. This direct extraction ensures a more faithful representation of the system’s dynamic behavior, providing a highly informative and noise-resilient spectral feature set known as matrix pencil mean frequency (MPMF). To further enhance robustness and simulate real-world industrial environments, additive white Gaussian noise (AWGN) was introduced at both the signal and feature levels, creating diversified datasets with varying signal-to-noise ratios (SNRs). This dual augmentation strategy significantly improved the generalization ability of the proposed system. Validation experiments conducted on the University of Ottawa’s UORED-VAFCLS dataset demonstrated that the MPMF-MLP framework achieves outstanding performance, maintaining high classification accuracy even in harsh noisy conditions where traditional methods typically degrade. Furthermore, the proposed MPMF-MLP model exhibits strong adaptability under dynamic operating conditions such as variable speed and load, owing to the inherent capability of the Matrix Pencil Method to capture transient frequency variations directly from non-stationary signals. This property allows the extracted features to remain physically meaningful and discriminative despite fluctuations in operating parameters. As a result, the system can maintain reliable fault diagnosis performance across different industrial regimes, demonstrating its potential for deployment in practical, time-varying environments. Overall, the proposed method offers clear advantages in terms of noise robustness, accuracy, and adaptability. It represents a significant advancement in machinery condition monitoring and fault diagnosis, providing a scalable and practical solution for real-time industrial applications. In this study, data augmentation was deliberately performed using AWGN to introduce controlled variability in the vibration and feature domains. This approach provides a physically interpretable and reproducible means of simulating the noisy conditions typically encountered in industrial machinery. While more sophisticated generative models such as GANs or VAEs could further diversify the data, their synthetic outputs may not always reflect realistic vibration behavior, potentially affecting diagnostic interpretability. Future research will explore hybrid data augmentation strategies that combine noise-based and generative techniques to enhance dataset diversity while maintaining the physical integrity of vibration signals. In addition, subsequent studies will focus on validating the proposed approach under variable-speed and variable-load conditions and investigating its integration into predictive maintenance frameworks to enable intelligent fault prognosis in real-world industrial applications. Also the present study employed a controlled sensor configuration consistent with the University of Ottawa UORED-VAFCLS dataset to ensure experimental repeatability. While this setup offers a reliable benchmark for evaluating algorithmic performance, it may not capture all practical variations in industrial sensor placement or mounting conditions. Nevertheless, the proposed MPMF method operates solely on the spectral content of vibration signals, making it inherently adaptable to different measurement configurations. Future work will focus on validating this adaptability across multiple sensor locations and machine types to confirm its suitability for real-world industrial deployment.
